# Determining prognostic variables of treatment outcome in obsessive–compulsive disorder: effectiveness and its predictors in routine clinical care

**DOI:** 10.1007/s00406-021-01284-6

**Published:** 2021-07-03

**Authors:** Philipp Herzog, Bernhard Osen, Christian Stierle, Thomas Middendorf, Ulrich Voderholzer, Stefan Koch, Matthias Feldmann, Winfried Rief, Eva-Lotta Brakemeier

**Affiliations:** 1 Department of Clinical Psychology and Psychotherapy, Philipps-University of Marburg, Gutenbergstraße 18, 35032 Marburg, Germany; 2 Schön-Klinik Bad Bramstedt, Psychosomatic Clinic, Birkenweg 10, 24576 Bad Bramstedt, Germany; 3 Schön-Klinik Bad Arolsen, Psychosomatic Clinic, Hofgarten 10, 34454 Bad Arolsen, Germany; 4 Schön-Klinik Roseneck, Psychosomatic Clinic, Am Roseneck 6, 83209 Prien am Chiemsee, Germany

**Keywords:** Obsessive–compulsive disorder, Effectiveness, Predictors, Patient characteristics, Prognostic variables

## Abstract

The objectives of this study were to investigate the naturalistic effectiveness of routine inpatient treatment for patients with obsessive–compulsive disorder (OCD) and to identify predictors of treatment outcome. A routinely collected data set of 1,596 OCD inpatients (*M* = 33.9 years, SD = 11.7; 60.4% female) having received evidence-based psychotherapy based on the cognitive–behavioral therapy (CBT) in five German psychotherapeutic clinics was analyzed. Effect sizes (Hedges' g) were calculated for several outcome variables to determine effectiveness. Predictor analyses were performed on a subsample (N = 514; *M* = 34.3 years, SD = 12.2; 60.3% female). For this purpose, the number of potential predictors was reduced using factor analysis, followed by multiple regression analysis to identify robust predictors. Effect sizes of various outcome variables could be classified as large (*g* = 1.34 of OCD–symptom change). Predictors of changes in OCD and depressive symptoms were symptom severity at admission and general psychopathological distress. In addition, patients with higher social support and more washing compulsions benefited more from treatment. Subgroup analyses showed a distinct predictor profile of changes in compulsions and obsessions. The results indicate that an evidence-based psychotherapy program for OCD can be effectively implemented in routine inpatient care. In addition to well-established predictors, social support, and washing compulsions in particular were identified as important positive predictors. Specific predictor profiles for changes in obsessions and compulsions are discussed.

**Supplementary Information:**

The online version contains supplementary material available at 10.1007/s00406-021-01284-6.

## Introduction

Obsessive–compulsive disorder (OCD) is a serious and debilitating disease [1, 2]. Patients suffering from OCD are usually treated with cognitive–behavioral therapies (CBT) which usually refer to exposure with response prevention (ERP), cognitive therapy (CT), and combinations thereof. CBT have been shown to be very effective, with large standardized effect sizes (ES) ranging from 1.31 compared with waiting list, 1.33 compared with placebo conditions, and 0.55 compared with antidepressant medication [3]. No significant differences have been found between ERP and CT [3], suggesting that they alleviate symptoms in OCD patients to the same extent. In general, a combination of CBT and medication appears to be no more effective than CBT plus a placebo [3], although better results can be achieved for severe OCD with a combination treatment [4].

These findings lead to the conclusion that ERP and CT can be considered the “gold standard” in the treatment of OCD. However, although CBT is considered a first line, evidence-based psychological treatment in OCD, its integration into daily clinical practice is hampered by several problems, e.g., patients’ lack of motivation, limited numbers of clinicians qualified to provide it, and treatment delivery challenges such as alleged organizational difficulties [5, 6]. This research-practice gap is supported by studies showing that less than half of therapists regularly use ERP [7] and about 21% of psychotherapists trained in CBT never or rarely use ERP in the outpatient treatment of OCD [8]. In line with this, from the patients’ perspective, two fifths of OCD patients in one study reported that they had never received CBT with ERP [9]. Thus, the consequent use of ERP in an inpatient setting appears to be a promising approach, since a higher treatment dose can be delivered by several therapists.

Throughout Germany, inpatient treatment is provided to OCD patients who are considered to be the most severely treatment-resistant—a difficult-to-treat population—and for whom no outpatient treatment is available. However, the question of whether CBT for OCD can be effectively used in routine clinical care has not been fully clarified. One study has shown that CBT in a routine clinical inpatient setting can achieve large standardized effect sizes for multimorbid, pretreated OCD patients [10]. Furthermore, the mean dropout rate from OCD treatment in controlled studies is about 15% [11], with CT exhibiting the lowest rate (11.4%) and ERP (19.1%) and combined ERP and antidepressant medication (32.0%) showing the highest [3]. Refusal rates vary widely, depending on the study [3]. Even where patients receive the “gold standard,” about 30% appear not to benefit from treatment in terms of nonresponse [12]. Only 50–60% of OCD patients experience clinically meaningful changes, while about 75% report residual symptoms [13]. Therefore, in addition to the above-mentioned effectiveness question, it would seem promising to identify stable predictors of treatment outcome (i.e., prognostic variables) to determine patients at risk for nonresponse.

Two reviews have identified some important predictors which, at first glance, lead to similar results. In the first, symptom severity, symptom subtype, severe depression, presence of comorbid personality disorders, family dysfunction, and the therapeutic alliance were found to be among the most consistent and important predictors [14]. The other review predicted poorer treatment outcomes as a result of hoarding pathology, higher anxiety, and symptom severity, certain symptom subtypes, unemployment, and being single/not married [15]. Similar findings were reported by Keeley et al. (2008).

In contrast with the above-mentioned reviews, original studies sometimes produce different results because they investigate different variables as predictors, which in turn have an influence on the selection of studies in meta-analyses and systematic reviews, e.g., limitations due to selective inclusion and reporting of outcomes and analyses [16]. Of note, meta-analyses are prone to biases both at the level of individual trials and the dissemination of trial results [17], e.g., “garbage-in-garbage-out” problem (i.e., inclusion of poor quality studies) and “apple-with-oranges” problem (i.e., mixing of dissimilar studies) that has been identified as a potential validity threat among other general limitations, such as publication bias [18]. A detailed summary of the literature on predictors of treatment outcome in OCD can be found in the Supplemental material 1. In summary, a number of studies have identified specific predictors of outcome, but to date no comprehensive models of robust predictors of treatment outcome that include sociodemographic, clinical, and psychological variables have been identified. Of note, the concept of treatment-resistant/treatment-refractory OCD [19, 20] might be in part a negative mirror of predictors of beneficial treatment response (i.e., indicating nonresponse). Although a significant number of studies report a relationship between demographic and clinical variables and treatment outcome, the results are to some extent inconsistent, leading to the conclusion that reliable evidence on predictors of treatment outcome in OCD is still lacking (for a detailed overview of the current literature, we refer to Supplemental Material 1).

The main limitations of previous studies are their lack of power due to small sample sizes, and their focus on either sociodemographic or clinical or psychological variables alone. Also, the research indicates that reducing the pool of potential predictors prior to data analyses might be useful because an increased number of predictors could lead to more uncertainty regarding an individual predictor, especially but not limited to standard linear regression models [21, 22]. In this study, we try to overcome both these shortcomings. Therefore, the aims of the current explorative study are: (1) to determine the effectiveness of inpatient OCD treatment in routine clinical care; and (2) to examine the predictive relationship of sociodemographic, clinical, and psychological variables in a single model of treatment outcome using novel statistical approaches to reduce the number of variables.

## Methods

This study was conducted in accordance with the ethical standards as laid down in the 1964 Declaration of Helsinki and its subsequent amendments.

### Sample

For this study, we were able to analyze data routinely collected between 2013 and 2017 from OCD inpatients treated in five German clinics offering specialized inpatient treatment. In the German mental healthcare system, specialized inpatient treatment is given to OCD patients when outpatient treatment fails, or is not available. The patients in these clinics completed various self-report questionnaires at admission and at discharge and were interviewed by raters. All patients gave informed consent to anonymous evaluations of their routinely collected data. The present study included all patients consecutively diagnosed with OCD [F42 according to ICD-10; 23] by practitioners between 2013 and 2017, who met the following inclusion and exclusion criteria. The inclusion criteria were having a primary diagnosis of OCD, the focus of treatment being on OCD, and being 18–65 years of age. The exclusion criteria were having comorbid mental and behavioral disorders due to psychoactive substance use (F1 according to ICD-10) and/or comorbid schizophrenia, schizotypal, delusional, and other nonmood psychotic disorders (F2 according to ICD-10). In addition to the evaluation of a trained therapist, the reliability for an OCD diagnosis was assured using a cutoff in the Y-BOCS scores of at least 16 for F42.2 (according to ICD-10), and, respectively, at least 10 for F42.0 and F42.1 (according to ICD-10), and further reassured by the head of the unit. To deal with the heterogeneity of OCD patients, no further exclusion criteria were defined a priori.

**Fig. 1:**
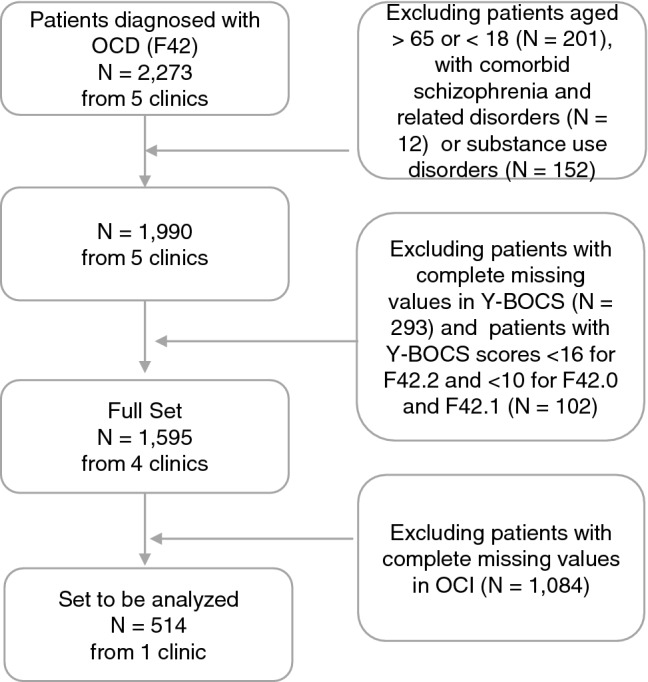
Patient flow diagram. For the sake of simplicity, we used the term “comorbid schizophrenia and related disorders” and “substance use disorders” in the exclusion criteria. Of note, these terms refer to comorbid schizophrenia, schizotypal, delusional, and other non-mood psychotic disorders (F2 according to ICD-10), and comorbid mental and behavioral disorders due to psychoactive substance use (F1 according to ICD-10), respectively

A total of 1595 consecutive patients from four clinics met the inclusion criteria and were used to calculate effect size. A subsample of only 514 patients from one clinic could be included in the final analysis, since the data on the relevant potential predictors were not available (in particular, there was no data on the Obsessive–Compulsive Inventory—Revised [OCI-R])^1^ for many of the patients. At first glance, it appeared that the subsample was different on several important sociodemographic and clinical variables (e.g., Y-BOCS baseline score, OCD subtype), as indicated by significant differences. However, most of these differences could be explained by the large sample size and further visual inspection revealed no clinically relevant differences. Thus, the subsample’s sociodemographic and clinical variables did not differ in a clinically meaningful manner from those of the full sample (see Table 1). The patient flow diagram is shown in Fig. 1.

**Table 1:** Sample characteristics of the full sample (*n* = 1595) and OCI-R (*n* = 514) subsample

Characteristics	OCI-R sample (*n* = 514)	Full sample (*n* = 1595)	*p*
Age at admission *M* (SD)	34.3 (12.2)	33.9 (11.7)	.304
Sex *n* (%)			.986
Male	204 (39.7)	631 (39.6)	
Female	310 (60.3)	964 (60.4)	
Educational score* M* (SD) ^a^	3.3 (0.8)	3.3 (0.8)	.862
OCD subtype *n* (%)^b^			< .001
Predominantly obsessional thoughts (F42.0)	28 (5.4)	101 (6.3)	
Predominantly compulsive actions (F42.1)	128 (24.9)	243 (15.2)	
Mixed thoughts and actions (F42.2)	354 (68.9)	1247 (78.2)	
Unspecified (F42.9)	4 (0.8)	4.0 (0.3)	
Number of mental comorbidities *M* (SD)^b^	1.4 (1.0)	1.3 (1.0)	.002
Most frequent mental comorbidities *n* (%)^b^			
Depressive episode (F32)	142 (27.6)	491 (30.8)	.068
Recurrent depressive disorder (F33)	231 (44.9)	659 (41.3)	.049
Phobic disorder (F40)	73 (14.2)	188 (11.8)	.048
Personality disorder (F6)	81 (15.7)	224 (14.0)	.200
Housing situation *n* (%)			< .001
Alone	125 (20.4)	220 (13.8)	
Co-living with partner or family	269 (44.0)	474 (29.7)	
Co-living with parents	31 (5.1)	321 (20.1)	
Institutional placement	4 (.7)	10 (0.6)	
Shared flat, private flat, furnished room	162 (26.5)	509 (31.9)	
No fixed household	1 (.3)	8 (0.5)	
Flat-sharing community	18 (2.9)	29 (1.8)	
Marital status *n* (%)			< .001
Married	184 (35.8)	493 (30.9)	
Divorced	15 (2.9)	65 (4.1)	
Widowed	4 (0.1)	5 (0.3)	
Single	311 (60.5)	1032 (64.7)	
In a relationship *n* (%)	251 (48.9)	790 (49.6)	
Occupational status *n* (%)			.357
Unemployed	125 (25.1)	374 (23.5)	
Retired	57 (11.4)	173 (10.9)	
Student, in training, home care	99 (19.9)	328 (20.6)	
Working full time	152 (30.5)	498 (30.7)	
Working half time	43 (8.6)	144 (9.0)	
Working occasionally	19 (3.8)	41 (2.6)	
Ability to work *n* (%)	235 (45.7)	708 (44.5)	.523
First inpatient treatment *n* (%)	319 (74.7)	927 (79.1)	.006
Outpatient psychotherapy *n* (%)	373 (72.6)	1033 (64.8)	< .001
Outpatient psychiatric treatment *n* (%)	343 (67.0)	1000 (62.9)	.022
Y-BOCS score at baseline *M* (SD) ^c^	24.7 (5.5)	25.5 (5.6)	< .001
OCI-R mean score at baseline *M* (SD) ^d^	30.2 (12.3)	30.2 (12.3)	
PHQ-9 score at baseline *M* (SD) ^e^	12.5 (6.0)	12.7 (6.0)	.425
GAF score at baseline *M* (SD) ^f^	45.7 (9.4)	45.7 (8.5)	.976
BSI mean score at baseline *M* (SD) ^g^	1.35 (.7)	1.3 (0.7)	.103
SWLS score at baseline *M* (SD) ^h^	14.7 (6.8)	15.1 (6.8)	.132

*M* = mean, *SD* = Standard deviation, *n* = number; *p* = p value from tests for difference between samples with and without OCI values in full set; *t* test for continuous variables, chi-squared-test for dichotomous variables and Fisher’s test for categorical variables with more than two levels

^a^Based on the German school system; scale from 0 (no degree) to 4 (general qualification for university entrance)

^b^Diagnosis as given by practitioners according to ICD-10

^c^Y-BOCS = Yale-Brown Obsessive–Compulsive Scale: 10 items, scale 0−40

^d^OCI-R = Obsessive–Compulsive Inventory-Revised: 18 items, scale 0−72

^e^PHQ-9 = Patient Health Questionnaire-9: 9 items scale 0−27

^f^GAF = Global Assessment of Functioning: Scale 0−100

^g^BSI = Brief Symptom Inventory: 53 items, scale 0−4

^h^Satisfaction With Life Scale: 5 Items, scale 5−35

Relevant sociodemographic and clinical characteristics of the full and the OCI-R sample are displayed in Table 1. Exemplarily, the relevant sociodemographic and clinical features of the OCI-R sample are described below:

The OCI-R sample consisted of 514 patients with a mean age of 34.3 years (SD = 12.2), 60.3% of whom were female. Almost half of the samples were co-habiting with a partner or family (44.0%), about one quarter shared a flat (26.5%), and one fifth lived alone (20.4%). The mean education score according to the German school system was 3.3, indicating a relatively highly educated patient sample. Although 60.5% patients were not married, nearly half (48.9%) were in a permanent relationship. One quarter (25.1%) were unemployed, 30.5% patients worked full time, and only 45.7% were able to work. For one-third (33.9%), this was their first inpatient treatment. Before beginning this inpatient treatment, 72.6% had one kind of outpatient psychotherapy and 67.0% had one outpatient psychiatric treatment.

According to the ICD-10 and regarding OCD subtype a, 68.9% of the sample had mixed thoughts and actions. On average, patients exhibited 1.4 mental comorbidities, depressive disorders being among the most frequent. The Y-BOCS mean score was 24.7 and the OCI-R score 30.2, both indicating a moderate to severely disabled sample. In addition, the PHQ-9 mean score was 12.5, indicating moderate depressive symptoms, while general psychopathology was high as indicated by a GSI (mean BSI score) of 1.35 (SD = 0.70) compared with the mean GSI score of inpatient psychiatric patients (*M* = 1.19; SD = 0.86) [24]. A mean GAF score of 45.7 at admission indicated severely impaired general functioning as a result of serious symptoms. The overall satisfaction with life as measured by the SWLS was rather low, at 14.7.

### Routine clinical care treatment

The multiple clinics are part of one large clinic group and all offer a multimodal symptom-specific CBT treatment program for OCD based on the national and international guidelines of CBT for OCD [25, 26]. These guidelines also refer to modern transdiagnostic approaches like Acceptance Commitment Therapy [ACT; 27]. In Germany, outpatient treatments for OCD very often fall short of offering the necessary amount and quality of therapist-accompanied ERPs [7, 9]. Therefore, OCD-specific inpatient treatment programs aim to deliver a maximum of therapist-accompanied ERPs as a crucial change factor in the treatment of OCD. The inpatient treatment in these clinics is carried out on specific wards for the treatment of OCD (i.e., in community with other OCD inpatients), and comprises three stages in individual and group therapy: ‘motivation and psychoeducation’ (1–2 weeks), ‘exposure with response prevention (ERP)’ (4–6 weeks) and ‘transfer and relapse prevention’ (1–2 weeks). The therapeutic elements of this CBT treatment program are as follows:

The first stage of ‘psychoeducation’ includes OCD-specific behavior analyses based on the cognitive–behavioral model of OCD [28], the identification of individual safety behavior and avoidance strategies, the rationale of expositions, the development of an individual hierarchy of critical situations and cognitive therapy (e.g., with focus on the appraisal of individual thoughts as threatening, probability bias and thought–action fusion) as well as a detailed functional assessment of OCD [e.g., 29]. In the second stage of ‘exposure with response prevention (ERP)’, patients receive a minimum of three accompanied ERPs within the individual psychotherapy plus a minimum of four ERPs delivered by specifically trained nurses in individual- or group setting and weekly protocol groups including behavior analyses of critical situations and review of self-controlled ERPs. In addition, about 2 h per day are reserved for self-controlled ERPs, behavioral training of norm behavior accompanied by therapeutic contracts defining ‘reference dates’ to strengthen commitment to norm behavior after expositions, and behavioral training of alternative coping strategies. In the third stage of ‘transfer and relapse prevention’, the focus lies on home exposition [including videoconference-based therapeutic supervision; cf. 30], inclusion of relatives and partners into individual psychotherapy and peer-support groups with former OCD patients in remission.

Multimodal and transdiagnostic interventions include weekly interactional ward groups (2 h per week; e.g., elaboration of alternative social skills, and coping with emotions, clarification of ‘normal’ behavior, identification, and treatment of individual functions of OCD), weekly mindfulness groups (1 h per week), sports, and body therapy groups (3 h per week) as well as access to social skills training and/or symptom-specific group therapy for comorbid disorders (e.g., depression or social phobia) (2 h per week), and relaxation training, art therapy group, biofeedback and social counseling (by trained social workers) (2 h per week) according to individual indication and psychiatric comorbidities. In addition, modules of ACT with a focus on the application of acceptance-based strategies on coping with obsessions and compulsive behavior are included into individual and group therapies (2 h per week in group therapy). Therapy is delivered under weekly team supervision and a weekly visit of a senior physician.

In total, patients receive at least 1 h per week individual psychotherapy, 7 h per week specific OCD therapy plus a minimum of 12 h per week of multimodal and transdiagnostic interventions over 8–10 weeks leading to a total of 160–200 therapy hours.

In our sample, the average length of stay was *M* = 54.88 days (SD = 18.02) for the full sample and *M* = 65.84 days (SD = 22.50) for the OCI-R sample.

For the patient-staff ratio on each OCD ward, a minimum of two licensed psychotherapists with specific training in OCD treatment are available (3–5 years of training in CBT; one medical doctor and one certified clinical psychologist), 1–2 therapists in advanced CBT training, and at least 1, 5 certified nursing staff member, through which the adherence to OCD-specific behavior therapy including therapist-accompanied ERPs could be guaranteed. During the weekends, emergency care of the clinic with medical and nursing personal is warranted.

Psychopharmacological treatment was administered when needed and indicated, mainly to treat psychiatric comorbidities, in compliance with the current national and international guidelines for OCD treatment and in accordance with clinical expert supervision [25, 26]. Nevertheless, the major treatment focus lied on psychotherapeutic work in single and group settings; medication was therefore continued or reduced to former psychopharmacological treatment before intake.

Medication in eight classes of substances (namely, antidepressants, neuroleptics, tranquilizers, anticonvulsants, narcotics, substitution, analgesics/antiphlogistics, and other medications) was recorded in the data set only at discharge. In the full sample, the proportional distribution is as follows: antidepressants (*n* = 986, 61.8%), neuroleptics (*n* = 273, 17.1%), tranquilizers (*n* = 23, 1.4%), anticonvulsants (*n* = 24, 1.5%), narcotics (*n* = 7, 0.4%), substitution (*n* = 20, 1.3%), analgesics/antiphlogistics (*n* = 135, 8.5%), other medication (*n* = 521, 32.7%), no medication (*n* = 303, 19.0%), unknown/unclear (*n* = 41, 2.6%). In the OCI-R sample, the proportional distribution of the medication is as follows: antidepressants (*n* = 288, 56.0%), neuroleptics (*n* = 49, 9.5%), tranquilizers (*n* = 2, 0.4%), anticonvulsants (*n* = 3, 0.6%), narcotics (*n* = 3, 0.6%), substitution (*n* = 3, 0.6%), analgesics/antiphlogistics (*n* = 19, 3.7%), other medication (*n* = 84, 16.3%), no medication (*n* = 111, 21.6%), unknown/unclear (*n* = 28, 5.4%).

## Measures

Primary treatment outcome in terms of symptom-specific change was assessed at admission and discharge using the following measures: the Yale-Brown Obsessive–Compulsive Scale (Y-BOCS) and the revised Obsessive–Compulsive Inventory (OCI-R). As for secondary treatment outcomes, the following measures were assessed at admission and discharge: Beck’s Depression Inventory-II (BDI-II), the Brief Symptom Inventory (BSI), the Patient Health Questionnaire-9 (PHQ-9), the Patient Health Questionnaire-15 (PHQ-15), the General Anxiety Disorder-7 (GAD-7), the General Assessment of Functioning (GAF), and the Satisfaction with Life Scale (SWLS). For the symptom-specific primary outcome Y-BOCS-SR and the secondary outcome PHQ-9, prediction analyses were computed.

### Primary outcome: obsessive–compulsive symptoms

The Yale-Brown Obsessive Compulsive Scale (Y-BOCS) [31] was originally developed as a clinician-rated interview measuring the severity of obsessive–compulsive disorder. It consists of 10 items, each rated from 0 (no symptoms) to 4 (extreme symptoms). Separate subscores for the severity of obsessions and compulsions can be calculated. A self-rating scale of the Y-BOCS (Y-BOCS-SR) was developed; while one study showed only a moderate relationship between the interview and the self-report version of the Y-BOCS in a clinical sample of OCD patients [32], another study has shown excellent internal consistency, test–retest reliability, and convergent validity [33].

For further analyses, the revised Obsessive–Compulsive Inventory (OCI-R) [34] was used. The OCI-R is a self-report instrument measuring various symptom domains of obsessive–compulsive disorder. It contains 18 items over six subscales: washing, checking, ordering, obsessing, hoarding, neutralizing. Items are rated on a five-point Likert-scale indicating the distress caused by symptoms, ranging from 0 (not at all) to 4 (extremely). The OCI-R has shown good to excellent internal consistency, test–retest reliability, and convergent reliability [34].

### Secondary outcomes: depression, anxiety, somatic symptoms, psychological distress, general functioning, quality of life

The Patient Health Questionnaire (PHQ-D) is a self-administered questionnaire consisting of the PHQ-9 assessing depression, the GAD-7 measuring generalized anxiety disorder, and the PHQ-15 measuring somatic symptoms. In addition, the data from the BSI [35], BDI-II [36], GAF [37], and SWLS [38] were used in the analyses. Among all secondary outcomes, the PHQ-9 was chosen for predictor analyses due to the high rate of comorbid depression in this sample and fewer missing data as compared to the BDI-II.

### Other measures

At admission, patients answered sociodemographic questions in a self-report questionnaire covering age, sex, number of children, marital status, living conditions, ability to work, wish to retire, and so forth. In addition, clinical characteristics such as mental and medical diagnoses, previous psychotherapeutic and/or psychiatric treatment, and number of comorbidities were assessed at this stage.

### Statistical analysis

All statistical analyses were conducted in the statistical processing language R [39]. Time-event data were log-transformed. Univariate outlier values were removed by a *p* < .001 criterion [40] adapted to the large sample size on the *z* distribution for all continuous variables, post-treatment values of the outcomes, and change scores. Missing data imputation was performed by random forest imputation using the R package *missForest* [41], a nonparametric imputation algorithm based on the random forests that results in fewer imputation and prediction errors when compared with other commonly used imputation techniques [42].

To determine the effectiveness of routine clinical care treatment, unstandardized effect sizes for the full and the OCI-R sample were calculated using Hedge’s g. In addition, despite criticism of its use in clinical trials [43], the Last Observation Carried Forward (LOCF) method was used as a more conservative method for estimating effect size. Since this study was naturalistic in nature, LOCF seemed to be a satisfactory way of comparing standardized effect sizes (i.e., as compared to controls) with uncorrected effect sizes (i.e., based on the raw pre–post differences) we computed to get a more holistic estimation of the real effect size.

To reduce the dimensionality of the data, sparse principal component analysis (SPCA) (*α* = 0.005) was performed on the predictors using the package *sparsepca* [44]. SPCA has emerged as a powerful data analysis technique with better interpretability than that of traditional principal component analysis (PCA). Because of ambiguous scree plot results, Horn’s parallel analysis [45] was performed with the function fa.parallel in the R package *psych* [46] to determine the optimal number of components. For each outcome (Y-BOCS-SR as primary and PHQ-9 as secondary), a stepwise multiple regression model was fitted. In the first step, the baseline sum score of the two outcomes was used as the single predictor. In the second step, the components derived from the SPCA were included as well.

## Results

### Effectiveness of the inpatient treatment

In the full sample, the mean difference between the Y-BOCS scores pretreatment (*M* = 25.5, SD = 5.3) and post-treatment (*M* = 16.0, SD = 7.2) was 9.5 points (SD = 7.1). For the primary treatment outcome, Hedge’s g of the symptom change score in the Y-BOCS total was 1.34 (95% CI [1.26, 1.43]) while the LOCF-corrected Hedge’s g was 1.10 (95% CI [1.03, 1.17]), indicating large effect sizes. Even with the LOCF-corrected estimation, effect sizes for the Y-BOCS pre-/postchange remained large. With regard to the subscales, Hedge’s g of the symptom change in Y-BOCS behavior was 1.29 (95% CI [1.21, 1.37]), while the LOCF-corrected ES was 1.10 (95% CI [1.00, 1.15]); Hedge’s g of the symptom change in Y-BOCS thoughts was to some extent lower, i.e., 1.10 (95% CI [1.00, 1.18]), and for LOCF-corrected 0.93 (95% CI [0.85, 1.00]).

In the OCI-R sample, the pretreatment (*M* = 24.7, SD = 5.5) and post-treatment scores (*M* = 15.6, SD = 7.0) were slightly lower than in the full sample, as was the mean difference between pre- and post-treatment scores (*M* = 9.0, SD = 7.2). The unstandardized effect sizes of all outcomes are shown in Table 2.

**Table 2:** Means (M), standard deviations (SD), and effect sizes (ES) at pre- and post-treatment for the OCR-I subsample (*N* = 514)

	Pre		Post		*t*	*p*	|ES|	95% CI |ES|
	*M*	SD	*M*	SD				
OCI-R total	1.68	0.68	0.98	0.63	23.80	< .001	1.17	1.02; 1.31
Washing	2.06	1.48	1.15	1.14	16.87	< .001	0.84	0.70; 0.99
Obsessing	2.60	1.18	1.72	1.16	16.72	< .001	0.83	0.69; 0.98
Hoarding	0.88	1.05	0.53	0.80	9.11	< .001	0.46	0.32; 0.60
Ordering	1.55	1.34	0.91	10.11	13.53	< .001	0.67	0.53; 0.82
Checking	1.99	1.33	1.05	1.00	18.66	< .001	0.92	0.78; 1.06
Neutralizing	0.94	1.12	0.51	0.82	10.26	< .001	0.52	0.38; 0.67
Y-BOCS total	24.66	5.53	15.55	7.04	25.77	< .001	1.25	1.10; 1.40
Compulsions	2.57	0.64	1.58	0.80	25.07	< .001	1.20	1.05; 1.34
Obsessions	2.42	0.72	1.60	0.81	19.88	< .001	0.99	0.84; 1.13
BDI-II	26.54	11.40	15.03	11.15	19.86	< .001	1.12	0.95; 1.29
GSI (BSI)	121.22	60.80	77.24	55.99	18.24	< .001	0.86	0.72; 1.00
PHQ-9	12.52	5.98	7.85	5.23	18.11	< .001	0.86	0.72; 1.00
GAD-7	1.59	0.66	0.97	0.62	22.17	< .001	1.06	0.92; 1.20
PHQ-15	0.74	0.40	0.54	0.35	14.66	< .001	0.70	0.56; 0.83
GAF	45.69	9.42	58.78	11.67	27.21	< .001	1.24	1.38; 1.10
SWLS	14.70	6.81	18.15	6.99	12.08	< .001	0.57	0.71; 0.44

OCI-R = Obsessive Compulsive Inventory-Revised; Y-BOCS = Yale-Brown Obsessive–Compulsive Scale; BDI-II = Beck Depression Inventory-II; GSI (BSI) = Global Severity Index of the Brief Symptom Inventory; PHQ-9 = Patient Health Questionnaire-9; GAD-7 = General Anxiety Disorder-7; PHQ-15 = Patient Health Questionnaire-15; SWLS = Satisfaction with Life Scale; GAF = Global Assessment of Functioning

Even in the OCI-R sample, the effect sizes with respect to the change in Y-BOCS symptoms could be considered large: Hedge’s g of the symptom change score in the OCI-R was 1.17 (95% CI [1.02, 1.31]), which indicates large effects overall, while the subscales showed differences. Most of the effect sizes in subscales were large, with two exceptions: Hedge’s g of symptom changes in OCI-R hoarding 0.46 (95% CI [0.32, 0.60]) and neutralizing 0.52 (95% CI [0.38, 0.67]) could be considered medium. The effect sizes of other secondary outcomes ranged between medium for the SWLS and PHQ-15 to large for the BDI-II, PHQ-9, BSI (GSI), GAD-7, and GAF (see Table 2).

In addition, we analyzed (partial) response and remission in the full and the OCI-R sample using the information stated by an international expert consensus for defining treatment response, remission, recovery, and relapse in OCD [47]. In the full sample, response (without CGI criterion) was achieved by *n* = 686 (43.0%) and response (with CGI- criterion) by *n* = 530 (33.2%) patients, while in addition *n* = 211 (13.2%) patients reached partial response (without CGI criterion), and *n* = 201 (12.6%) partial response (with CGI criterion), respectively. In total, *n* = 424 (26.5%) patients have reached remission in the full sample. In the OCI-R sample, the rates were similarly distributed in percentage. Response (without CGI criterion) was reached by *n* = 209 (40.7%) and response (with CGI criterion) by *n* = 162 (31.5%) patients, while in addition *n* = 66 (12.8%) patients achieved partial response (without CGI criterion), and *n* = 62 (12.1%) partial response (with CGI criterion), respectively. In this subsample, remission was achieved by *n* = 142 (27.6%) patients. Restrictively, no data were available for the additional CGI-I criterion “lasting for at least one week” regarding (partial) response calculation and no information on the CGI-S was available for the additional criterion in remission.

### Dimensional reduction

The SPCA revealed 10 distinct factors to best represent the underlying data of 54 potential predictors. These ten factors, interpreted by the variables with the highest factor loading and considering the side loadings, were labelled as follows: “Distress,” “Somatic disorders,” “Obsessing,” “Social support,” “Ordering,” “Chronicity of depression,” “Comorbid depression,” “Academic,” “Functional disability,” and “Washing behaviour.” These factors were then used in the subsequent regression analyses.

The results of the SPCA, including factor loadings and communalities as well as the scree plot, are presented in the Supplemental material (see Supplemental material 2 and 3).

### Predictors of primary treatment outcome

The regression results for the Y-BOCS total score as the criterion are shown in Table 3, the regression results for the Y-BOCS subscores “Obsessions” and “Compulsions” in the Supplemental material 4.

**Table 3:** Regression results using Y-BOCS total score post-treatment as the criterion for the OCR-I subsample (N = 514)

Predictor	*beta*	*beta* 95% CI	*p*	*sr*^*2*^	*sr*^*2*^ 95% CI	*r*	Fit *R*^*2*^	Difference Δ*R*^*2*^
(Intercept)								
Baseline	0.37**	[0.28, 0.45]	< .001	0.13	[NA, NA]	0.37**		
							0.133**	
(Intercept)								
Baseline	0.33**	[0.21, 0.45]	< .001	0.06	[0.02, 0.10]	0.37**		
Distress	0.21**	[0.10, 0.32]	< .001	0.03	[– 0.00, 0.05]	0.33**		
Somatic disorders	0.03	[– 0.06, 0.12]	.512	0.00	[– 0.00, 0.01]	0.11*		
Obsessing	0.04	[– 0.05, 0.13]	.417	0.00	[– 0.00, 0.01]	0.18**		
Social support	– 0.10*	[– 0.19, – 0.01]	.036	0.01	[– 0.01, 0.02]	– 0.07		
Ordering	0.07	[– 0.04, 0.17]	.201	0.00	[– 0.01, 0.01]	0.26**		
Chronic depression	0.01	[– 0.08, 0.10]	.825	0.00	[– 0.00, 0.00]	0.04		
Depression	0.01	[– 0.08, 0.10]	.792	0.00	[– 0.00, 0.00]	0.10*		
Academic	0.02	[– 0.07, 0.10]	.695	0.00	[– 0.00, 0.00]	0.03		
Disability	0.08	[– 0.01, 0.18]	.083	0.01	[– 0.01, 0.02]	0.23**		
Washing behavior	– 0.21**	[– 0.33, – 0.10]	< .001	0.03	[– 0.00, 0.05]	0.12*		
							0.220**	0.087**
								95% CI [0.04, 0.13]

A significant beta-weight indicates that semi-partial correlations are also significant. *beta* indicates the standardized regression weights. *sr*^2^ represents the semi-partial correlation squared. *r* represents the zero-order correlation

*Indicates *p* < 0.05

**Indicates *p* < 0.01

For the Y-BOCS total score post-treatment as the criterion, the baseline Y-BOCS score as predictor in the first step explained 13.3% of the variance and had a significant effect (*β* = 0.37, *p* < .001). The second set of predictors added another 8.7% of explained variance (95% CI [0.04, 0.13], *p* < .01), so that the overall 22% of the variance could be explained. In this step and additionally for the Y-BOCS baseline score (*β* = 0.33, *p* < .001), the factors “distress” (*β* = 0.21, *p* < .001), “social support” (*β* = − 0.10, *p* = .036), and “washing behavior” (*β* = − 0.21, *p* < .001) had significant effects.

The baseline Y-BOCS Obsessions score as predictor in the first step explained 20.6% of the variance and significantly predicted Y-BOCS Obsessions scores post-treatment (*β* = 0.45, *p* < .001). The second series of predictors contributed another 8.8% of the explained variance (95% CI [0.04, 0.13], *p* < .01), so that the overall 29.4% of the variance could be explained. In this step and additionally for the Y-BOCS Obsessions baseline score (*β* = 0.37, *p* < .001), the factors “distress” (*β* = 0.24, *p* < .001), “obsessing” (*β* = 0.14, *p* = .002), and “washing behavior” (*β* = – 0.24, *p* < .001) had significant effects on Y-BOCS Obsessions scores post-treatment.

For the Y-BOCS Compulsions score post-treatment as the criterion, the baseline Y-BOCS Compulsions score as predictor in the first step explained a significant proportion of variance (13.0%) and had a significant effect (*β* = 0.36, *p* < .001). The second group of predictors added another 5.9% of the explained variance (95% CI [0.02, 0.10], *p* < .01), so that the overall 18.9% of the variance could be explained. In this step and additionally for the Y-BOCS Compulsions baseline score (*β* = 0.32, *p* < .001), the factors “distress” (*β* = 0.12, *p* = .035) and “ordering” (*β* = 0.12, *p* = .028) significantly predicted Y-BOCS Compulsion scores post-treatment.

All other potential predictors had no significant effect either on the total score or on the scores of the subscales post-treatment (*p* > .05).

### Predictors of secondary treatment outcome

The regression results for the PHQ-9 score post-treatment as the criterion are listed in the Supplemental material 5. The baseline PHQ-9 score as predictor in the first step explained 32% of the variance and had a significant effect on PHQ-9 score post-treatment (*β* = 0.57, *p* < .001). Another 7.1% of the explained variance was added by the second set of predictors (95% CI [0.03, 0.11], *p* < .01), so that the overall 39.1% of the variance could be explained. In this step and additionally for the PHQ-9 baseline score (*β* = 0.28, *p* < .001), the factors “distress” (*β* = 0.31, *p* < .001), “social support” (*β* = – 0.12, *p* = .002), and “disability” (*β* = 0.12, *p* = .003) significantly predicted PHQ-9 scores post-treatment. All other potential predictors had no significant effect (*p* > .05).

## Discussion

This study sheds some light on the routine clinical care of OCD patients: first, by determining the effectiveness of an inpatient OCD treatment on symptom-specific as well as functional outcomes under everyday health care conditions; and secondly, by identifying important predictors of treatment outcome in a large sample of OCD patients being treated in German clinics offering specialized inpatient psychotherapy. Encouragingly, the overall large unstandardized, but corrected effect sizes indicate that OCD treatment is delivered effectively in routine clinical inpatient care, despite the fact that the pre-/posteffect sizes were marginally lower than those reported in other studies [48] and a COCHRANE review [49]. Notwithstanding this, given the naturalistic nature of the study this finding speaks in favor of the widespread implementation of effective OCD treatment in daily practice in Germany. Concerning the effectiveness vs. efficacy discussion [cf. 50], the effect sizes found in this study could be interpreted as being very good. Owing to the large and well-described inpatient OCD sample, the corrected effect sizes might be seen as a benchmark for OCD treatment. Furthermore, a number of relevant predictors were identified, replicating and building on earlier research results [51–56] through a study combining a large, well-described sample, novel state-of-the-art statistical procedures, and the inclusion of sociodemographic, clinical, and psychometric variables in a single model.

### Main findings and clinical implications

The sample in this study seems to be typical of a German inpatient clinic: in another sample from two German inpatient clinics, a similar mean score for age, gender distribution, and Y-BOCS was reported, albeit with a slightly higher OCI-R score and a higher percentage of comorbid personality disorders [57]. However, Voderholzer and colleagues’ sample did not include patients with a comorbid depressive disorder, while a high percentage of our study’s sample showed depressive comorbidity. Differences between the pretreatment and post-treatment Y-BOCS scores were slightly lower than the mean difference of 10.7 points (95% CI: 9.8–11.5) computed in a recent meta-analysis [58]. However, as pretreatment OCD severity appears to be a predictor of treatment outcome [58], the lower difference scores might be a result of relatively less OCD severity when compared with the meta-analytic mean. This could, in turn, be partly a result of using a cutoff score of 10 for the Y-BOCS on diagnosis F42.0 and F42.1 instead of an overall cutoff score of 16.

Overall, and in line with the previous studies [51–54], pretreatment OCD symptom severity and general distress appear to predict post-treatment symptom severity. In general, for symptom-specific change, OCD patients with lower general distress, higher social support, and higher scores on washing behavior appear to benefit most from the treatment.

Two conclusions might be drawn from this result: First, a lack of social support appears to diminish treatment outcome. This finding is in line with the results of a recent systematic review [55] in which the authors concluded that negative social support appeared to be associated with more severe symptoms whereas positive social support could be beneficial for OCD patients. This is also in accordance with another study that highlighted the quality of social support [59]. Hence, our findings once again underscore the role of relatives in supporting OCD patients recovering from their illness. There is no doubt that living with a patient who suffers from severe OCD can be a challenge for family members, as the compulsions can manifest themselves in daily interactions and affect relationships. In particular, living together can become a burden if relatives do not know how to deal with such behavior. It could therefore be beneficial either to involve relatives of OCD patients in the treatment process by providing them with useful information, e.g., psychoeducation on how to deal with the disease and its symptoms in everyday life, or to strengthen the quality of relationships through systemic or family interventions [60]. This might also lead to a reduction in comorbid depressive symptoms, as our results highlight the positive role of social support in reducing depressive symptoms in OCD patients.

Second, our analyses indicate that, surprisingly, OCD patients who exhibit more washing behavior benefit more from treatment than do other subgroups of OCD patients. This may be because washing behavior during CBT (respectively ERP) is both easier to treat and treatable in the inpatient setting when compared with other compulsive behaviors, such as hoarding which is more a problem in patients’ daily lives at home, or checking which is sometimes difficult to stop in patients’ minds. Future studies conducted in inpatient settings could provide more evidence for this finding.

Subanalyses revealed distinct predictor profiles for each of the symptom-specific reductions (obsessions and compulsions): interestingly, the results suggest that OCD patients with higher scores on obsessing thoughts appear to benefit less from inpatient treatment in terms of a reduction in obsessions. The relatively short CBT treatment involved may not be sufficient to reduce pure obsessions. Hence, for patients with high scores on obsession scales it may be useful to integrate some metacognitive elements [61] from metacognitive therapy [62, 63] into the CBT to improve their treatment outcome. Recently, several research groups have applied metacognitive theories to OCD [64–68]. On the other hand, OCD patients with higher scores on washing behavior appear to benefit more from the treatment through a reduction in their obsessions. This result adds to the evidence base for CBT in treating OCD patients with high levels of washing behavior, and stresses the crucial role of changing debilitating obsessions in such patients.

Also noteworthy is the finding that OCD patients with higher scores on ordering appear to benefit less from the treatment in terms of reducing compulsions. For these patients, traditional CBT treatment may not be effective in reducing their compulsions. Practitioners might therefore consider incorporating other elements, such as home visits, into treatment planning, as has been suggested in specialized CBT treatment for patients with compulsive hoarding [69].

### Strengths and limitations

A major strength of the current study is the large sample size, combined with the use of the “gold-standard” of disorder-specific self-report measures for OCD, i.e., the Y-BOCS-SR, as the primary outcome. In addition, the sample was well characterized by sociodemographic and clinical data. Furthermore, the dimensional reduction of sociodemographic, clinical, and psychological variables to interpretable factors was rendered “state-of-the-art” by using a data-driven, novel, statistical technique—sparse principal component analysis—which proved to be superior to previous factor analysis techniques.

Within a phase IV study, our study exhibits high external validity while internal validity aspects might be compromised. The interpretation of the results is therefore limited by the naturalistic and thus uncontrolled and nonrandomized nature of the study, i.e., the lack of a control group and no assessment of treatment integrity nor patient engagement/compliance. The treatment selection was not random, so unknown patient and/or clinician characteristics that contributed to treatment choice may have been relevant. Since there was no strict CBT treatment manual, practitioners may have chosen different techniques (possibly no CBT-specific strategies) to treat the specific needs of individual patients. However, one of the co-authors (UV) was scientifically responsible for the adequate implementation of the current national guidelines of OCD treatment [25, 26], in which CBT and ERP in particular were the main components throughout the data collection period. On the other hand, one of the strengths of a naturalistic design is its closer approximation to clinical practice and therefore higher external validity—even if this is at the expense of internal validity. Furthermore, other important therapeutic factors probably contributed to treatment outcome. As a limitation, we have neither information on the number of therapists, their competence, their level of training or their therapeutic experience, nor data on therapist variables and the therapeutic relationship were collected: However, therapist effects seem to play an important role [70–72] and explain about 5% of the variability of the treatment outcome [73]. Notably, these effects were mostly larger in the naturalistic setting than in clinical trials [74]. Therefore, it is even more important to control for this in naturalistic settings, especially in inpatient psychotherapy, as therapist effects seem to be greater with more severely ill patients [75]. This seems to be due to their ability to handle interpersonally challenging encounters with patients, i.e., their facilitative interpersonal skills [76]. A recent review demonstrated that more effective therapists are characterized by professionally cultivated interpersonal capacities that are likely rooted in their personal lives and attachment histories [77]. Another important aspect of therapeutic factors that can contribute to treatment outcome concerns the therapeutic relationship [78]. Indeed, the design of the therapeutic relationship is an important therapeutic factor that seems to have an influence on the treatment outcome in a variety of contexts [79–84]. In particular, the correlation between the "real" relationship and treatment outcome [85] and the influence of rupture repair in the therapeutic relationship on treatment outcome have also been demonstrated [86]. Moreover, therapist variability seems to predict treatment outcome in the context of the therapeutic relationship [87]. Another major limitation is the lack of data on medication and psychopharmacological interventions. As such, we were not able to analyze potential beneficial effects of concomitant pharmacotherapy and its interaction effects with psychotherapy on treatment outcome as only medication data at discharge were available. Future routine clinical care studies should record medication at intake to control for this aspect. Moreover, this study lacks long-term/follow-up outcome assessment, which could be of importance because of the controlled nature of the residential environment. As a further limitation, the design and evaluation of this study was based solely on retrospectively collected data, in which we were unable to include any measurement instruments other than those mentioned above. Relatedly, this study relied on self-report measures [32], i.e., Y-BOCS-SR and OCI-R, which were used because they are cost-effective to use in daily patient care. This is insofar problematic as it reduces the reliability with which the diagnosis of OCD was assigned. That aside, various outcomes were considered, and the findings of the Y-BOCS-SR were supported by the results of the OCI-R. However, future studies should also include observer-rated instruments in large samples, e.g., by developing new, cost-effective, therapist-rated instruments to assess OCD symptoms. Lastly, there is a significant loss of patients from effectiveness analysis to predictor analyses. Yet, the authors decided to include the OCI-R as a crucial and potentially informative candidate predictor.

### Future research directions

With regard to naturalistic studies, future research could include routine outcome monitoring [ROM; 88, 89] with the aim of further benchmarking OCD treatments, predicting early changes [early responder, sudden gains; 90, 91], and correcting negative developments [sudden losses; 92]. Despite the promising findings of this study, it remains a future research question whether these predictors, more specifically prognostic variables, can be replicated under more standardized conditions (such as RCTs). Therefore, these predictors should be validated in prospective controlled studies, focusing on long-term outcomes to build on the findings presented here. In addition, there may be other important predictors that were not included in this study, e.g., the therapeutic relationship [93], motivational processes [94–96], and patient adherence [97, 98], childhood maltreatment [99], and expectations [100]. Future studies should routinely collect data on therapist variables (e.g., experience as indicated by years of training, level of competence as indicated by interpersonal skill set) and the therapeutic relationship using adequate measures. For example, the Working Alliance Inventory [WAI; 101] is the most frequently used measure to assess the therapeutic relationship that has also been validated for the use with patients with severe mental illness in psychiatric settings [102], followed by the Helping Alliance Questionnaire [103] and other instruments (for an overview, see [104]). Finally, to guide treatment selection procedures, it is necessary to identify not only prognostic but also prescriptive variables that indicate the relative efficacy of one treatment over another [105]. Such research, which is also referred to as "practice-oriented research” or “patient-focused research” [106] and includes process measures, has become more and more a focus of interest in psychotherapy research, whereby large datasets from routine care, sometimes in the context of ROM, are used to generate useful statements that can be applied directly in clinical practice. The aim is to provide therapists with helpful feedback information to facilitate the decision-making process before and during the course of treatment, and thus to be able to treat patients in a more personalized and thus effective way.

## Supplementary Information

Below is the link to the electronic supplementary material.

Supplementary file1 (DOCX 37 KB)

Supplementary file2 (DOCX 25 KB)

Supplementary file3 (PNG 10 KB)

Supplementary file4 (DOCX 22 KB)

Supplementary file5 (DOCX 18 KB)

## Footnotes

1 This subsample is called the “OCI-R sample” in the following paragraphs.

## Declarations

### Data availability statement

Data sharing is not applicable to this article as only secondary analyses were performed on a routinely collected data set with permission to use for research purposes but without explicit permission of data sharing by the patients.
