# Supplementary material for: Determining prognostic variables of treatment outcome in obsessive–compulsive disorder: effectiveness and its predictors in routine clinical care
Source: Eur Arch Psychiatry Clin Neurosci. 2021 Jul 3;272(2):313–26. doi: 10.1007/s00406-021-01284-6 (PMC8866294; doi:10.1007/s00406-021-01284-6)
Supplement: Supplementary file 1 — Supplementary file1 (DOCX 37 KB) [file 406_2021_1284_MOESM1_ESM.docx]

**Sociodemographic Variables as Predictors**

The literature on important sociodemographic predictors appears sparse and need to be validated by further studies. However, with regard to such variables, being married appears to positively predict treatment outcome (Steketee, Eisen, Dyck, Warshaw, & Rasmussen, 1999). In line with this, in another study being married or cohabiting consistently predicted better outcomes in an inpatient setting (Boschen, Drummond, Pillay, & Morton, 2010). Other studies have argued that this result depends on the quality of social support: patients who perceived their relatives to be either critical or hostile had more severe OCD symptoms (Van Noppen & Steketee, 2009).

**Clinical Variables as Predictors**

**Symptom Severity.** With regard to important clinical variables, lower OCD symptom severity at admission has consistently been found across studies to be a positive predictor of treatment outcome (Farrell & Boschen, 2011; Kempe et al., 2007; Kyrios, Hordern, & Fassnacht, 2015; Raffin, Guimarães Fachel, Ferrão, Pasquoto de Souza, & Cordioli, 2009; Steketee et al., 1999, 2011). Moreover, higher scores on psychoticism, the presence of obsessions, and high general psychological distress have been found to be predictors of non-response (Kohls, Bents, & Pietrowsky, 2002).

**OCD subtypes and comorbid disorders.** Furthermore, some inconsistencies occur when other relevant clinical variables (for example, OCD subtype and the presence of comorbid Axis I and Axis II diagnoses) are examined more closely.

Hoarding symptoms are consistently found to be predictors of worse treatment outcomes (Abramowitz, Franklin, Schwartz, & Furr, 2003; Eisen et al., 2013; Maher et al., 2012; Veale et al., 2016), and in the long run too (Jakubovski et al., 2013). Yet, while one study found that the presence of sexual obsessions predicted poorer treatment response (Farrell & Boschen, 2011), another indicated conversely that the presence of sexual (but not religious) OCD symptoms predicted improvement (Steketee et al., 2011).

Some studies have found that more Axis I comorbid diagnoses (mainly major depressive disorder and anxiety disorders) and more depressive symptoms in general are linked to a higher chance of improvement (Kempe et al., 2007; Steketee et al., 2011). In contrast, OCD patients with higher depressive symptom severity have been found to show a moderate treatment outcome but significantly less improvement compared with that of less depressed patients (Abramowitz & Foa, 2000; Abramowitz, Franklin, Street, Kozak, & Foa, 2000). In accordance with this, the presence of at least one comorbid disorder and a depressive disorder also appears to predict poorer long-term outcomes (Jakubovski et al., 2013). In particular, patients with generalized anxiety disorder (GAD) and/or panic disorder comorbidity showed worse treatment outcomes post-treatment (Hansen, Vogel, Stiles, & Gunnar Götestam, 2007). Similarly, GAD was also found to be a predictor for dropout, whereas major depressive disorder (MDD) and all three personality clusters predicted worse post-treatment outcomes, as did MDD and GAD in the longer-term (Steketee, Chambless, & Tran, 2001). An obsessive-compulsive personality disorder (OCPD) diagnosis and greater OCPD severity was also observed to predict worse treatment outcomes (Eisen et al., 2013; Pinto, Liebowitz, Foa, & Simpson, 2011) as well as positive schizotypal symptoms did (Moritz et al., 2004). Conversely, other studies have found that personality disorders generally do not influence treatment outcome (Dreessen, Hoekstra, & Arntz, 1997) or long-term treatment outcome (Fricke et al., 2006). Another study also found that neither the presence nor the number of pre-treatment comorbid disorders predicted symptom severity, treatment response, remission, or clinically significant change post-treatment (Storch et al., 2010). And finally, in another comorbid alcohol misuse appeared to predict worse outcomes (Veale et al., 2016). In summary, the role of OCD subtypes, comorbid disorders (Axis I and II), and in particular depressive symptoms, as positive or negative predictors remains contentious and consistent findings lacking.

**Chronicity and Onset.** Looking at other clinical variables (for example, chronicity and onset), a longer duration of illness (Eisen et al., 2013) has been found to predict worse treatment outcomes, while early age of onset has been linked to a higher chance of remission (Kempe et al., 2007). One study identified different predictors of treatment outcome in early and late OCD (EOCD, LOCD): in EOCD, high motivation and high initial symptom severity were associated with better treatment outcomes, while higher age, longer duration of psychiatric inpatient treatment before assessment, and low level of social functioning were associated with worse outcomes; for LOCD, living in a stable relationship, high motivation, and completing treatment predicted better treatment outcomes, while a low level of psychological functioning and a longer duration of inpatient psychiatric treatment before assessment was associated with more undesirable outcomes (Langner et al., 2009).

**Obsessions vs. Compulsions.** Moreover, closer inspection suggests a pronounced predictor profile of poorer outcomes in obsessions and compulsions: greater initial severity of complaints and depression predicted worse outcomes for compulsions; while greater initial severity of complaints, higher levels of depression, longer problem duration, poorer motivation for treatment, and dissatisfaction with the therapeutic relationship predicted worse outcomes for obsessions (Keijsers, Hoogduin, & Schaap, 1994).

**References**

Abramowitz, J. S., & Foa, E. B. (2000). Does comorbid major depressive disorder influence outcome of exposure and response prevention for OCD? *Behavior Therapy*, *31*(4), 795–800. https://doi.org/10.1016/S0005-7894(00)80045-3

Abramowitz, J. S., Franklin, M. E., Schwartz, S. A., & Furr, J. M. (2003). Symptom Presentation and Outcome of Cognitive-Behavioral Therapy for Obsessive-Compulsive Disorder. *Journal of Consulting and Clinical Psychology*, *71*(6), 1049–1057. https://doi.org/10.1037/0022-006X.71.6.1049

Abramowitz, J. S., Franklin, M. E., Street, G. P., Kozak, M. J., & Foa, E. B. (2000). Effects of comorbid depression on response to treatment for obsessive-compulsive disorder. *Behavior Therapy*, *31*(3), 517–528. https://doi.org/10.1016/S0005-7894(00)80028-3

Boschen, M. J., Drummond, L. M., Pillay, A., & Morton, K. (2010). Predicting outcome of treatment for severe, treatment resistant OCD in inpatient and community settings. *Journal of Behavior Therapy and Experimental Psychiatry*, *41*(2), 90–95. https://doi.org/10.1016/j.jbtep.2009.10.006

Dreessen, L., Hoekstra, R., & Arntz, A. (1997). Personality disorders do not influence the results of cognitive and behavior therapy for obsessive compulsive disorder. *Journal of Anxiety Disorders*, *11*(5), 503–521. https://doi.org/10.1016/S0887-6185(97)00027-3

Eisen, J. L., Sibrava, N. J., Boisseau, C. L., Mancebo, M. C., Stout, R. L., Pinto, A., & Rasmussen, S. A. (2013). Five-year course of obsessive-compulsive disorder: Predictors of remission and relapse. *The Journal of Clinical Psychiatry*, *74*(3), 233–239. https://doi.org/10.4088/JCP.12m07657

Farrell, L. J., & Boschen, M. (2011). Treatment outcome in adult OCD: Predictors and processes of change. *Asia Pacific Journal of Counselling and Psychotherapy*, *2*(1), 82–97. https://doi.org/10.1080/21507686.2010.536915

Fricke, S., Moritz, S., Andresen, B., Jacobsen, D., Kloss, M., Rufer, M., & Hand, I. (2006). Do personality disorders predict negative treatment outcome in obsessive–compulsive disorders? A prospective 6-month follow-up study. *European Psychiatry*, *21*(5), 319–324. https://doi.org/10.1016/j.eurpsy.2005.03.010

Hansen, B., Vogel, P. A., Stiles, T. C., & Gunnar Götestam, K. (2007). Influence of Co‐Morbid Generalized Anxiety Disorder, Panic Disorder and Personality Disorders on the Outcome of Cognitive Behavioural Treatment of Obsessive‐Compulsive Disorder. *Cognitive Behaviour Therapy*, *36*(3), 145–155. https://doi.org/10.1080/16506070701259374

Jakubovski, E., Diniz, J. B., Valerio, C., Fossaluza, V., Belotto-Silva, C., Gorenstein, C., … Shavitt, R. G. (2013). Clinical predictors of long-term outcome in obsessive-compulsive disorder. *Depression and Anxiety*, *30*(8), 763–772. https://doi.org/10.1002/da.22013

Keijsers, G. P. J., Hoogduin, C. A. L., & Schaap, C. P. D. R. (1994). Predictors of Treatment Outcome in the Behavioural Treatment of Obsessive-Compulsive Disorder. *British Journal of Psychiatry*, *165*(06), 781–786. https://doi.org/10.1192/bjp.165.6.781

Kempe, P. T., van Oppen, P., de Haan, E., Twisk, J. W. R., Sluis, A., Smit, J. H., … van Balkom, A. J. L. M. (2007). Predictors of course in obsessive-compulsive disorder: logistic regression versus Cox regression for recurrent events. *Acta Psychiatrica Scandinavica*, *116*(3), 201–210. https://doi.org/10.1111/j.1600-0447.2007.00997.x

Kohls, S., Bents, H., & Pietrowsky, R. (2002). Prädiktoren für Erfolg und Misserfolg in der verhaltenstherapeutischen Behandlung von Zwangspatienten. *Verhaltenstherapie*, *12*(2), 98–106. https://doi.org/10.1159/000064373

Kyrios, M., Hordern, C., & Fassnacht, D. B. (2015). Predictors of response to cognitive behaviour therapy for obsessive-compulsive disorder. *International Journal of Clinical and Health Psychology*, *15*(3), 181–190. https://doi.org/10.1016/j.ijchp.2015.07.003

Langner, J., Laws, M., Röper, G., Zaudig, M., Hauke, W., & Piesbergen, C. (2009). Predicting Therapy Outcome in Patients with Early and Late Obsessive-Compulsive Disorder (EOCD and LOCD). *Behavioural and Cognitive Psychotherapy*, *37*(05), 485. https://doi.org/10.1017/S1352465809990294

Maher, M. J., Wang, Y., Zuckoff, A., Wall, M. M., Franklin, M., Foa, E. B., & Simpson, H. B. (2012). Predictors of patient adherence to cognitive-behavioral therapy for obsessive-compulsive disorder. *Psychotherapy and Psychosomatics*, *81*(2), 124–126. https://doi.org/10.1159/000330214

Moritz, S., Fricke, S., Jacobsen, D., Kloss, M., Wein, C., Rufer, M., … Hand, I. (2004). Positive schizotypal symptoms predict treatment outcome in obsessive–compulsive disorder. *Behaviour Research and Therapy*, *42*(2), 217–227. https://doi.org/10.1016/S0005-7967(03)00120-7

Pinto, A., Liebowitz, M. R., Foa, E. B., & Simpson, H. B. (2011). Obsessive compulsive personality disorder as a predictor of exposure and ritual prevention outcome for obsessive compulsive disorder. *Behaviour Research and Therapy*, *49*(8), 453–458. https://doi.org/10.1016/j.brat.2011.04.004

Raffin, A. L., Guimarães Fachel, J. M., Ferrão, Y. A., Pasquoto de Souza, F., & Cordioli, A. V. (2009). Predictors of response to group cognitive-behavioral therapy in the treatment of obsessive-compulsive disorder. *European Psychiatry : The Journal of the Association of European Psychiatrists*, *24*(5), 297–306. https://doi.org/10.1016/j.eurpsy.2008.12.001

Steketee, G., Chambless, D. L., & Tran, G. Q. (2001). Effects of axis I and II comorbidity on behavior therapy outcome for obsessive-compulsive disorder and agoraphobia. *Comprehensive Psychiatry*, *42*(1), 76–86. https://doi.org/10.1053/comp.2001.19746

Steketee, G., Eisen, J., Dyck, I., Warshaw, M., & Rasmussen, S. (1999). Predictors of course in obsessive compulsive disorder. *Psychiatry Research*, *89*(3), 229–238. https://doi.org/10.1016/S0165-1781(99)00104-3

Steketee, G., Siev, J., Fama, J. M., Keshaviah, A., Chosak, A., & Wilhelm, S. (2011). Predictors of treatment outcome in modular cognitive therapy for obsessive-compulsive disorder. *Depression and Anxiety*, *28*(4), 333–341. https://doi.org/10.1002/da.20785

Storch, E. A., Lewin, A. B., Farrell, L., Aldea, M. A., Reid, J., Geffken, G. R., & Murphy, T. K. (2010). Does cognitive-behavioral therapy response among adults with obsessive–compulsive disorder differ as a function of certain comorbidities? *Journal of Anxiety Disorders*, *24*(6), 547–552. https://doi.org/10.1016/j.janxdis.2010.03.013

Van Noppen, B., & Steketee, G. (2009). Testing a conceptual model of patient and family predictors of obsessive compulsive disorder (OCD) symptoms. *Behaviour Research and Therapy*, *47*(1), 18–25. https://doi.org/10.1016/j.brat.2008.10.005

Veale, D., Naismith, I., Miles, S., Gledhill, L. J., Stewart, G., & Hodsoll, J. (2016). Outcomes for residential or inpatient intensive treatment of obsessive-compulsive disorder: A systematic review and meta-analysis. *Journal of Obsessive-Compulsive and Related Disorders*, *8*, 38–49. https://doi.org/10.1016/j.jocrd.2015.11.005
