# Supplementary material for: Determining prognostic variables of treatment outcome in obsessive–compulsive disorder: effectiveness and its predictors in routine clinical care
Source: Eur Arch Psychiatry Clin Neurosci. 2021 Jul 3;272(2):313–26. doi: 10.1007/s00406-021-01284-6 (PMC8866294; doi:10.1007/s00406-021-01284-6)
Supplement: Supplementary file 2 — Supplementary file2 (DOCX 25 KB) [file 406_2021_1284_MOESM2_ESM.docx]

Table 2

*Factor loadings and communalities for the clinical sample (N=514): pattern matrix.*

| Variable | Factor | | | | | | | | | | | | | | Communality |
| --- | --- | --- | --- | --- | --- | --- | --- | --- | --- | --- | --- | --- | --- | --- | --- |
|  | Distress | | Somatic Disorders | | Obsessing | Social Support | | Ordering | Chronicity Depression | | Comorbid Depression | Academic | Functional Disability | Washing Behavior |  |
| Age |  | .03 | |  | | .50 | .09 | | .01 |  | |  |  |  | .70 |
| Gender |  |  | |  | |  | -.15 | |  |  | |  |  | .46 | .39 |
| First inpatient treatment |  |  | | -.01 | |  | .03 | |  |  | |  | -.06 | .04 | .06 |
| Ability to work |  |  | |  | |  |  | |  |  | |  | **-.59** |  | .58 |
| Outpatient psychotherapy |  |  | |  | | .03 | -.16 | |  |  | | -.05 |  |  | .10 |
| Psychiatric treatment |  |  | | .02 | |  |  | |  | .09 | |  | .27 | -.04 | .22 |
| Children |  |  | |  | | **.56** |  | |  |  | |  |  |  | .68 |
| SWLS |  |  | | .01 | | .19 | -.10 | |  |  | |  | -.07 | -.12 | .40 |
| BSI somatization | .18 | .01 | |  | |  |  | |  |  | |  |  |  | .53 |
| BSI obsession compulsion | .15 |  | |  | |  | .11 | |  |  | |  |  |  | .67 |
| BSI interpersonal sensitivity | .27 |  | | -.03 | |  |  | |  |  | |  |  |  | .67 |
| BSI depression | .26 |  | |  | |  |  | |  |  | |  |  |  | .69 |
| BSI hostility | .21 |  | |  | |  |  | |  |  | |  |  |  | .51 |
| BSI phobic anxiety | .12 |  | | .02 | |  |  | |  |  | |  | .03 |  | .40 |
| BSI paranoid ideation | .23 |  | | -.12 | |  |  | |  |  | |  |  |  | .58 |
| BSI psychoticism | .31 |  | |  | |  |  | |  |  | |  |  |  | .69 |
| BSI pst | **.43** |  | |  | |  |  | |  |  | |  |  |  | .80 |
| BSI psdi | .37 |  | |  | |  |  | |  |  | |  |  |  | .69 |
| PHQ mood | .28 |  | |  | |  |  | |  |  | |  |  |  | .72 |
| PHQ anxiety | .26 |  | | .03 | |  |  | |  |  | |  |  |  | .67 |
| PHQ physical symptoms | .25 |  | |  | |  |  | |  |  | |  |  |  | .59 |
| BSI anxiety | .28 |  | |  | |  |  | |  |  | |  |  |  | .70 |
| Number of Diagnosis |  | **.61** | |  | |  |  | |  |  | |  |  |  | .75 |
| GAF |  | .05 | |  | |  |  | |  |  | |  | -.42 | -.06 | .40 |
| OCI-R washing |  |  | |  | |  |  | |  |  | |  |  | **.60** | .62 |
| OCI-R obsessing | .11 |  | | .32 | |  |  | |  |  | |  |  |  | .43 |
| OCI-R hoarding |  |  | |  | |  | .40 | |  |  | |  |  |  | .41 |
| OCI-R ordering |  |  | | -.02 | |  | **.50** | |  |  | |  |  |  | .59 |
| OCI-R checking |  |  | |  | |  | .43 | |  |  | |  | -.01 |  | .44 |
| OCI-R neutralizing |  |  | | .07 | |  | .30 | |  |  | |  |  |  | .32 |
| Y-BOCS Behavior |  |  | |  | |  | .16 | |  |  | |  |  | .56 | .63 |
| Y-BOCS Thoughts | .01 |  | | .18 | |  |  | |  | -.02 | |  | .13 | .07 | .29 |
|  |  | .23 | | -.07 | |  |  | |  |  | |  | .02 |  | .23 |
| Diagnosis F32.1 |  |  | |  | |  |  | | **-.69** | .17 | |  |  |  | .81 |
| Diagnosis F33.1 |  |  | |  | |  |  | | .65 | .20 | |  |  |  | .80 |
| Diagnosis E |  |  | |  | |  |  | |  | **.73** | |  |  |  | .80 |
| Diagnosis F40 |  | .01 | |  | |  |  | |  |  | |  | .16 |  | .10 |
| Diagnosis F42.1 |  |  | | -.57 | |  |  | |  |  | |  |  |  | .85 |
| Diagnosis F42.2 |  |  | | **.65** | |  |  | |  |  | |  |  |  | .83 |
| Diagnosis F6 |  |  | |  | | -.04 | .01 | |  |  | |  | .24 |  | .18 |
| Diagnosis G |  | .19 | |  | |  |  | |  |  | |  |  | .13 | .21 |
| Diagnosis I |  | .22 | |  | | .01 | .12 | |  |  | |  |  |  | .28 |
| Diagnosis M5 |  | .35 | |  | |  |  | |  |  | |  |  |  | .44 |
| Diagnosis M62.89 |  | .26 | | .06 | |  | -.09 | |  | .03 | |  |  |  | .29 |
| Diagnosis M |  | .49 | |  | |  |  | |  |  | |  |  |  | .68 |
| Diagnosis N |  |  | |  | |  |  | |  | -.56 | |  |  |  | .60 |
| Educational level |  |  | | .05 | |  | -.18 | |  | .12 | |  | -.12 | .04 | .23 |
| Living alone |  |  | | -.07 | | -.01 | .02 | |  |  | | .21 |  |  | .14 |
| Job trained |  |  | |  | |  |  | |  |  | | **-.66** |  |  | .74 |
| Academic Degree |  |  | |  | |  |  | |  |  | | **.66** |  |  | .74 |
| Unemployed |  |  | |  | | -.27 | .15 | |  |  | |  |  |  | .28 |
| Retired |  |  | | -.05 | | .13 |  | | .08 |  | |  | .37 |  | .41 |
| Married |  |  | |  | | .49 |  | |  |  | |  |  |  | .55 |
| In a relationship |  |  | |  | |  | .03 | | -.04 |  | |  |  | -.01 | .05 |

*Note.* Extraction method: sparse principal component analysis. BSI: Brief Symptom Inventory; pst: positive symptom total; psdi: positive symptom distress index; SWLS: Satisfaction With Live Scale; OCI-R: Obsessive Compulsive Inventory-Revised; Y-BOCS: Yale-Brown Obsessive Compulsive Scale (Self Report); GAF: General Assessment of Functioning.
