# Supplementary figures and images for: Determining prognostic variables of treatment outcome in obsessive–compulsive disorder: effectiveness and its predictors in routine clinical care
Source: Eur Arch Psychiatry Clin Neurosci. 2021 Jul 3;272(2):313–26. doi: 10.1007/s00406-021-01284-6 (PMC8866294; doi:10.1007/s00406-021-01284-6)

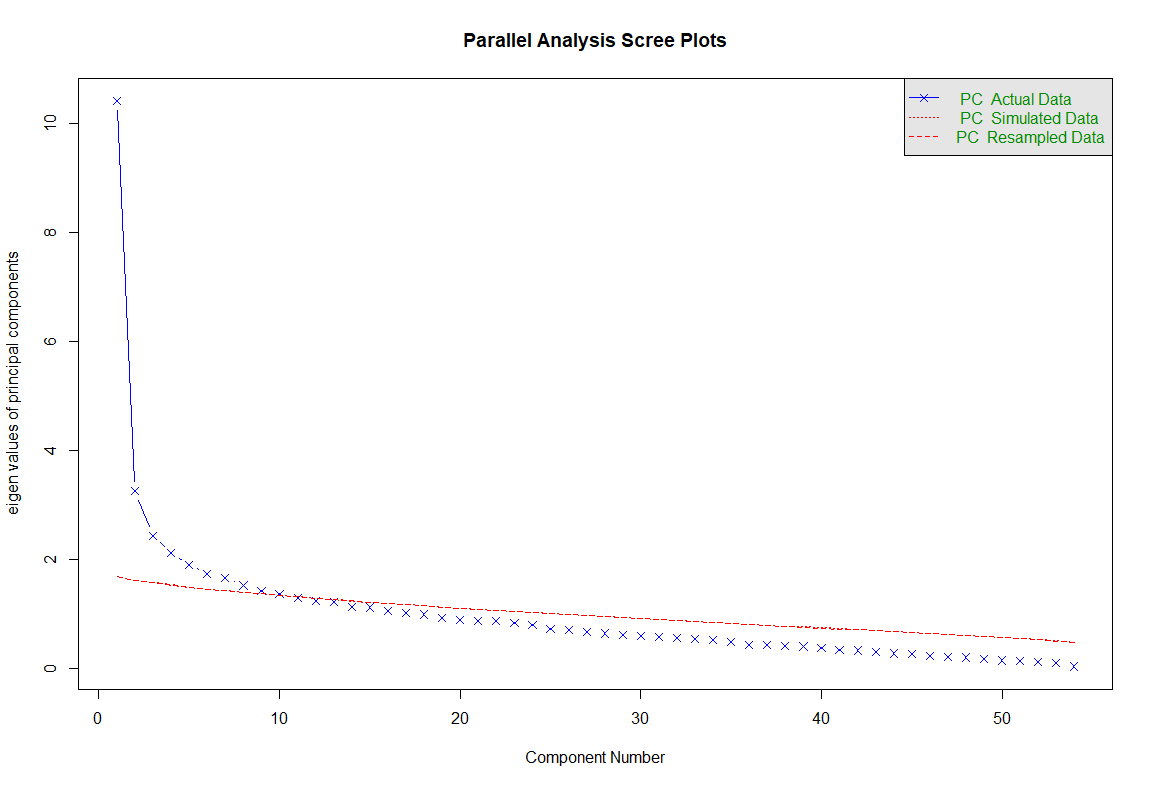

Supplement: Supplementary file 3 — Supplementary file3 (PNG 10 KB) [file 406_2021_1284_MOESM3_ESM.png]
