# Supplementary material for: Determining prognostic variables of treatment outcome in obsessive–compulsive disorder: effectiveness and its predictors in routine clinical care
Source: Eur Arch Psychiatry Clin Neurosci. 2021 Jul 3;272(2):313–26. doi: 10.1007/s00406-021-01284-6 (PMC8866294; doi:10.1007/s00406-021-01284-6)
Supplement: Supplementary file 4 — Supplementary file4 (DOCX 22 KB) [file 406_2021_1284_MOESM4_ESM.docx]

*Regression results using Y-BOCS sub-scores Obsessions and Compulsions post-treatment as the criterion for the OCR-I subsample (N = 514)*

| Y-BOCS Obsessions as criterion | | | | | | | | | | |
| --- | --- | --- | --- | --- | --- | --- | --- | --- | --- | --- |
| Predictor | *beta* | | *beta*  95% CI | | *p* | *sr^2^* | *sr^2^*  95% CI | *r* | Fit  *R^2^* | Difference  Δ*R^2^* |
| (Intercept) |  | |  | |  |  |  |  |  |  |
| Baseline | .45** | | [0.37, 0.54] | | <.001 | .21 |  | . 45** |  |  |
|  |  | |  | |  |  |  |  | .206** |  |
| (Intercept) |  | |  | |  |  |  |  |  |  |
| Baseline | .37** | | [0.27, 0.47] | | <.001 | .10 | [.05, .15] | .45** |  |  |
| Distress | .24** | | [0.14, 0.35] | | <.001 | .04 | [.01, .07] | .29** |  |  |
| Somatic disorders | .03 | | [-0.06, 0.12] | | .471 | .00 | [-.00, .01] | .10* |  |  |
| Obsessing | .14** | | [0.05, 0.23] | | .002 | .02 | [-.00, .04] | .30** |  |  |
| Social support | -.03 | | [-0.12, 0.05] | | .446 | .00 | [-.00, .01] | -.02 |  |  |
| Ordering | -.00 | | [-0.10, 0.09] | | .919 | .00 | [-.00, .00] | .12* |  |  |
| Chronic depression | -.00 | | [-0.08, 0.08] | | .999 | .00 | [-.00, .00] | .00 |  |  |
| Depression | -.01 | | [-0.09, 0.08] | | .879 | .00 | [-.00, .00] | .05 |  |  |
| Academic | .02 | | [-0.06, 0.11] | | .605 | .00 | [-.00, .00] | .02 |  |  |
| Disability | .06 | | [-0.04, 0.15] | | .241 | .00 | [-.01, .01] | .20** |  |  |
| Washing behavior | -.24** | | [-0.33, -0.14] | | <.001 | .04 | [.01, .07] | .01 |  |  |
|  |  |  | |  | |  |  |  | *.*294** | .088** |
|  |  |  | |  | |  |  |  |  | 95% CI [.04, .13] |

Y-BOCS Compulsions as criterion

| Predictor | *beta* | | *beta*  95% CI | | *p* | *sr^2^* | *sr^2^*  95% CI | *r* | Fit  *R^2^* | Difference  Δ*R^2^* |
| --- | --- | --- | --- | --- | --- | --- | --- | --- | --- | --- |
| (Intercept) |  | |  | |  |  |  |  |  |  |
| Baseline | 0.36** | | [0.27, 0.45] | | <.001 | .13 | [NA, NA] | .36** |  |  |
|  |  | |  | |  |  |  |  | .130** |  |
| (Intercept) |  | |  | |  |  |  |  |  |  |
| Baseline | 0.32** | | [0.19, 0.46] | | <.001 | .04 | [.01, .07] | .36** |  |  |
| Distress | 0.12* | | [0.01, 0.23] | | .035 | .01 | [-.01, .02] | .26** |  |  |
| Somatic disorders | 0.01 | | [-0.09, 0.10] | | .880 | .00 | [-.00, .00] | .08 |  |  |
| Obsessing | -0.08 | | [-0.17, 0.01] | | .083 | .01 | [-.01, .02] | -.02 |  |  |
| Social support | -0.09 | | [-0.18, 0.01] | | .065 | .01 | [-.01, .02] | -.07 |  |  |
| Ordering | 0.12* | | [0.01, 0.23] | | .028 | .01 | [-.01, .03] | .31** |  |  |
| Chronic depression | 0.03 | | [-0.06, 0.11] | | .550 | .00 | [-.00, .01] | .04 |  |  |
| Depression | 0.05 | | [-0.04, 0.14] | | .282 | .00 | [-.01, .01] | .13** |  |  |
| Academic | 0.00 | | [-0.09, 0.09] | | .992 | .00 | [-.00, .00] | .04 |  |  |
| Disability | 0.06 | | [-0.03, 0.15] | | .204 | .00 | [-.01, .01] | .17** |  |  |
| Washing behavior | -0.10 | | [-0.23, 0.04] | | .171 | .00 | [-.01, .01] | .24** |  |  |
|  |  |  | |  | |  |  |  | .198** | .059** |
|  |  |  | |  | |  |  |  |  | 95% CI [.02, .10] |
|  |  |  | |  | |  |  |  |  |  |

*Note.* A significant beta-weight indicates that semi-partial correlations are also significant. *beta* indicates the standardized regression weights. *sr^2^* represents the semi-partial correlation squared. *r* represents the zero-order correlation. * indicates *p* < .05. ** indicates *p* < .01.
