# Supplementary material for: Determining prognostic variables of treatment outcome in obsessive–compulsive disorder: effectiveness and its predictors in routine clinical care
Source: Eur Arch Psychiatry Clin Neurosci. 2021 Jul 3;272(2):313–26. doi: 10.1007/s00406-021-01284-6 (PMC8866294; doi:10.1007/s00406-021-01284-6)
Supplement: Supplementary file 5 — Supplementary file5 (DOCX 18 KB) [file 406_2021_1284_MOESM5_ESM.docx]

*Regression results using PHQ-9 score post-treatment as the criterion for the OCR-I subsample (N = 514)*

| Predictor | *beta* | | *beta*  95% CI | | *p* | *sr^2^* | *sr^2^*  95% CI | *r* | Fit  *R^2^* | Difference  Δ*R^2^* |
| --- | --- | --- | --- | --- | --- | --- | --- | --- | --- | --- |
| (Intercept) |  | |  | |  |  |  |  |  |  |
| Baseline | .57** | | [0.49, 0.64] | | <.001 | .32 |  | .57** |  |  |
|  |  | |  | |  |  |  |  | .320** |  |
| (Intercept) |  | |  | |  |  |  |  |  |  |
| Baseline | .28** | | [0.15, 0.42] | | <.001 | .06 | [.00, .05] | .57** |  |  |
| Distress | .31** | | [0.17, 0.44] | | <.001 | .03 | [.00, .05] | .57** |  |  |
| Somatic disorders | -.01 | | [-0.09, 0.07] | | .794 | .00 | [-.00, .00] | .09 |  |  |
| Obsessing | .01 | | [-0.07, 0.09] | | .778 | .00 | [-.00, .00] | .13** |  |  |
| Social support | -.12** | | [-0.20, -0.04] | | .002 | .01 | [-.00, .03] | -.12** |  |  |
| Ordering | .04 | | [-0.04, 0.13] | | .303 | .00 | [-.00, .01] | .29** |  |  |
| Chronic depression | .05 | | [-0.02, 0.13] | | .158 | .00 | [-.00, .01] | .09 |  |  |
| Depression | .00 | | [-0.08, 0.08] | | .967 | .00 | [-.00, .00] | .18** |  |  |
| Academic | -.02 | | [-0.10, 0.05] | | .592 | .00 | [-.00, .00] | -.00 |  |  |
| Disability | .12** | | [0.04, 0.20] | | .003 | .01 | [-.00, .03] | .29** |  |  |
| Washing behavior | -.08 | | [-0.16, 0.01] | | .073 | .03 | [-.01, .01] | .20** |  |  |
|  |  |  | |  | |  |  |  | *.391*** | .071** |
|  |  |  | |  | |  |  |  |  | 95% CI [.03, .11] |
|  |  |  | |  | |  |  |  |  |  |

*Note.* A significant beta-weight indicates that semi-partial correlations are also significant. *beta* indicates the standardized regression weights. *sr^2^* represents the semi-partial correlation squared. *r* represents the zero-order correlation. * indicates *p* < .05. ** indicates *p* < .01.
